# Plasma neurofilament light chain in type 2 diabetes: Associations with diabetic peripheral neuropathy, age, and renal function in a pilot cross-sectional study

**DOI:** 10.1371/journal.pone.0357207

**Published:** 2026-09-09

**Authors:** Michaela Škorvanová, Andrea Ižarik Verešpejová, Jozef Hanes, Radovan Plášil, Natália Huňarová, Jakub Šofranko, Li-Sheng Chien, Martin Kolísek

**Affiliations:** 1 Biomedical Centre Martin, Jessenius Faculty of Medicine in Martin, Comenius University in Bratislava, Martin, Slovakia; 2 Department of Medical Biochemistry, Jessenius Faculty of Medicine in Martin, Comenius University in Bratislava, Martin, Slovakia; 3 Institute of Neuroimmunology, Slovak Academy of Sciences, Bratislava, Slovakia; 4 Diabetology Outpatient Clinic, Vrútky, Slovakia; 5 Department of Molecular Biology, Faculty of Natural Sciences, Comenius University in Bratislava, Bratislava, Slovakia; University of Eastern Finland School of Medicine: Ita-Suomen yliopisto Laaketieteen laitos, FINLAND

## Abstract

**Background:**

Diabetic peripheral neuropathy (DPN) is a common complication of type 2 diabetes mellitus (T2DM). Neurofilament light chain (NfL) is a circulating marker of neuroaxonal injury, but its interpretation may be influenced by age and renal function.

**Objective:**

To evaluate the association between plasma NfL concentrations and DPN in T2DM while accounting for age and renal function.

**Methods:**

This pilot cross-sectional study included 41 patients with T2DM, of whom 20 had DPN diagnosed by a neurologist based on clinical and electrophysiological assessment and 21 had no DPN. Plasma NfL concentrations were measured using single-molecule array technology. Group differences and associations with clinical variables were assessed using non-parametric methods. Sequential linear regression models using natural log-transformed NfL evaluated the association of DPN status with NfL before and after adjustment for age and estimated glomerular filtration rate (eGFR).

**Results:**

Plasma NfL concentrations were higher in patients with DPN than in those without DPN (median 12.96 vs 8.29 pg/mL, p = 0.035). In sequential regression analysis, DPN status was associated with higher ln-transformed NfL in the unadjusted model (B = 0.499, 95% CI 0.108–0.890; p = 0.014), but the association was attenuated after adjustment for age and eGFR (B = 0.241, 95% CI −0.040–0.522; p = 0.091). In the fully adjusted model, age and eGFR remained associated with ln(NfL), and the model explained 62.0% of its variance.

**Conclusions:**

Plasma NfL concentrations were higher in patients with DPN in unadjusted analyses, but the association was attenuated after accounting for age and renal function. These exploratory findings highlight the importance of considering age and renal function when interpreting circulating NfL concentrations in T2DM and require confirmation in larger prospective cohorts.

## Introduction

Diabetic sensorimotor polyneuropathy (DPN) is the most common chronic complication of diabetes mellitus and affects up to half of patients with both type 1 and type 2 diabetes during the course of the disease [1]. Despite its high prevalence and substantial clinical burden, DPN frequently remains underdiagnosed, particularly in early or subclinical stages, because early manifestations may be subtle and diagnosis requires integration of clinical assessment with objective methods for evaluating peripheral nerve dysfunction [1–3]. Established objective diagnostic methods include nerve conduction studies for large-fiber dysfunction and dedicated assessments of small-fiber involvement, although their availability and application may vary across clinical settings [1,3].

Neurofilament light chain (NfL) is a neuron-specific structural protein of the axonal cytoskeleton and a major component of intermediate filaments expressed in both the central and peripheral nervous systems [4]. Following axonal injury or degeneration, NfL is released into the extracellular space and subsequently becomes detectable in cerebrospinal fluid and peripheral blood, making it a sensitive biomarker of neuroaxonal damage that can be measured in blood [4,5]. Over the past decade, circulating NfL concentrations have been extensively investigated across a wide range of neurological disorders, including neurodegenerative diseases, ischemic stroke, and traumatic brain injury, where they have been associated with neuroaxonal injury and clinically relevant measures of disease severity or outcome [6–8].

More recently, attention has focused on the role of NfL in peripheral neuropathies. Elevated serum or plasma NfL levels have been reported in hereditary and inflammatory peripheral neuropathies, with higher concentrations associated with measures of disease severity or activity [9–11]. Emerging evidence suggests that circulating NfL is associated with diabetic neuropathy across different study populations and neuropathy phenotypes [2,12–15]. A previous study identified NfL as a candidate biomarker of DPN [13], and subsequent cross-sectional studies reported associations between circulating NfL concentrations and diabetic neuropathy in adults with T2DM and individuals with youth-onset T2DM [12,14]. In addition, a longitudinal study demonstrated differences in serum NfL trajectories according to the development of early DPN [15], while a recent cross-sectional study showed associations between circulating NfL concentrations and structural and functional measures of nerve fiber loss in painful and painless DPN [2].

Beyond biochemical associations, recent studies have demonstrated correlations between circulating NfL concentrations and structural alterations of peripheral nerves assessed by ultrasonography, further supporting the link between NfL levels and morphological nerve damage in DPN [16]. Nevertheless, available evidence remains limited and partly heterogeneous, particularly with respect to study populations, neuropathy definitions, and study designs [2,12–15]. Moreover, circulating NfL concentrations are influenced by age and BMI [17], while renal function has also been associated with blood NfL concentrations [17–19], potentially complicating the interpretation of circulating NfL in patients with diabetes. Diagnostic definitions requiring nerve conduction abnormalities primarily identify neuropathy involving large myelinated fibers and may not capture patients with predominantly small-fiber neuropathy [1,3].

Therefore, the aim of this exploratory pilot cross-sectional study was to evaluate the association between plasma NfL concentrations and clinically and electrophysiologically supported DPN in patients with type 2 diabetes mellitus and to examine how adjustment for age and renal function influenced this association.

## Methods

### Study design and population

This pilot cross-sectional study included adults with type 2 diabetes mellitus (T2DM) treated at a diabetes outpatient clinic in Vrútky, Slovakia. Participants were consecutively enrolled during routine follow-up visits after ethics approval (March 27, 2025) until November 2025. Venous blood samples were collected at the time of enrollment. All participants provided written informed consent prior to inclusion in the study. Before study enrollment, all participants had undergone neurological examination and bilateral nerve conduction studies (NCS) of both lower legs performed by a neurologist as part of routine clinical care. Participants were stratified according to the presence or absence of DPN based on the diagnosis established by the neurologist before inclusion in the study. The approximately balanced group sizes resulted from consecutive enrollment and subsequent classification according to the pre-existing neurologist-established DPN diagnosis; no matching or quota-based sampling was performed.

### Inclusion and exclusion criteria

Eligible participants were adults with T2DM. In the enrolled cohort, the observed duration of T2DM ranged from 1 to 32 years. The diagnosis of T2DM was established according to the World Health Organization (WHO) criteria (1999). No upper age limit was specified for study eligibility.

Exclusion criteria included type 1 diabetes mellitus, significant hepatic or thyroid dysfunction, severe renal impairment (estimated glomerular filtration rate [eGFR] < 30 mL/min/1.73 m^2^, CKD stages G4–G5), uncontrolled arterial hypertension, pregnancy, abnormal complete blood count, recent infection, history of neurological disorders (including stroke), autoimmune diseases, or other potential causes of peripheral neuropathy.

### Diagnosis of diabetic peripheral neuropathy

Participants were classified as having DPN based on a diagnosis established by a neurologist before study enrollment as part of routine clinical care. The diagnosis was based on medical history, clinical neurological examination, and electrophysiological findings and required the presence of neuropathic sensory symptoms (e.g., tingling, burning, or stabbing pain in the toes, feet, or lower limbs), objectively reduced distal sensation, diminished or absent ankle reflexes, and pathological nerve conduction study (NCS) findings, after exclusion of other major causes of polyneuropathy. The requirement for both clinical manifestations and abnormal nerve conduction was consistent with the Toronto Consensus framework for confirmed diabetic sensorimotor polyneuropathy [3].

NCS examinations had been performed bilaterally in the lower limbs before study enrollment as part of routine neurological care. Electrophysiological abnormalities in at least two clinically relevant distal lower-limb nerves were required for classification as DPN. Participants with clinical symptoms or signs suggestive of neuropathy but without electrophysiological confirmation were not classified as having DPN in the present study. Because the neurological and electrophysiological assessments had been performed before study enrollment and were not conducted specifically for the purposes of the present study, detailed information on the individual nerves examined, specific sensory testing modalities, stimulation and recording distances, limb temperature monitoring, nerve-specific electrophysiological values, and sural nerve involvement was not available for retrospective analysis.

### Clinical, anthropometric and laboratory data

Anthropometric parameters were measured using standardized clinical procedures. Clinical and laboratory data, including diabetes duration, lipid profile (total cholesterol, LDL cholesterol, HDL cholesterol, triglycerides), glycated hemoglobin (HbA1c), estimated glomerular filtration rate (eGFR), and antidiabetic treatment, were obtained from medical records.

### Ethical approval

All participants provided written informed consent prior to inclusion and agreed to the use of pseudonymized data for research purposes. The study was approved by the Ethics Committee of Jessenius Faculty of Medicine, Comenius University in Martin (approval number EK 5/2025, March 27, 2025), and was conducted in accordance with the Declaration of Helsinki.

### Study measures

#### NfL analysis.

Venous blood samples were collected into BD Vacutainer® K2 EDTA tubes under standardized conditions. Plasma was separated by centrifugation at 4000 rpm for 10 minutes at 4°C. The obtained plasma was aliquoted (150 μL) and stored at −80°C until transport on dry ice to the Institute of Neuroimmunology, Slovak Academy of Sciences (Bratislava, Slovakia), where neurofilament light chain (NfL) concentrations were quantified. All samples underwent a single freeze–thaw cycle prior to analysis. The stability of NfL under these conditions has been previously validated [20,21]. Samples were blinded before laboratory analysis. Plasma NfL concentrations were measured in duplicates using ultrasensitive single-molecule array (Simoa) technology on a fully automated Simoa HD-1 Analyzer with the Quanterix Simoa® NF-Light Advantage PLUS Reagent Kit (Quanterix, Lexington, MA, USA), following the manufacturer’s protocol and previously published methodological standards [22,23]. Concentrations were calculated using proprietary Simoa™ HD-1 software.

### Statistical analysis

Continuous variables were assessed for normality using standard diagnostic methods (including visual inspection and normality testing) and are presented as median (interquartile range, IQR) due to non-normal distributions. Given the relatively small sample size and non-normality of several variables, a consistent non-parametric approach was applied.

The study used a between-subjects design comparing patients with diabetic peripheral neuropathy (DPN) and those without neuropathy (NoDPN). Between-group comparisons were performed using two-sided Mann–Whitney U tests for continuous variables and χ² tests or Fisher’s exact tests for categorical variables, as appropriate.

Associations between plasma neurofilament light chain (NfL) concentrations and clinical variables were evaluated using Spearman’s rank correlation coefficients, including analyses stratified by study group. The group-stratified analyses were exploratory and were intended to characterize associations within the two clinically defined groups rather than to formally test whether correlation coefficients differed between groups. Given the exploratory and hypothesis-generating nature of this pilot study, no correction for multiple comparisons was applied; therefore, these analyses should be interpreted with caution.

Sequential linear regression models were performed to evaluate the association between DPN status and plasma NfL concentrations and to examine how this association changed after adjustment for age and renal function. Because plasma NfL concentrations were right-skewed, NfL values were natural log-transformed, and ln(NfL) was used as the dependent variable in all regression models. NoDPN was used as the reference category for DPN status. Model 1 included DPN status as the sole explanatory variable. Model 2 included DPN status and age. Model 3, considered the fully adjusted model, included DPN status, age, and renal function, expressed as estimated glomerular filtration rate (eGFR). The number of explanatory variables was deliberately restricted because of the limited sample size and exploratory pilot design. Regression results are reported as unstandardized regression coefficients (B) with 95% confidence intervals (CIs) and p values. Model performance was assessed using the coefficient of determination (R^2^), adjusted R^2^, and the overall F-test. Multicollinearity was evaluated using variance inflation factors (VIF), with no evidence of significant multicollinearity observed.

Model assumptions were assessed by visual inspection of residual-versus-fitted plots and Q–Q plots of residuals, together with the Shapiro–Wilk test of residuals. The linearity of the associations of age and eGFR with ln(NfL) was additionally examined by adding centered quadratic terms for age and eGFR to the fully adjusted model. The extended model was compared with the primary fully adjusted model using a partial F-test. Neither quadratic term was statistically significant, and their joint inclusion did not improve model fit; therefore, age and eGFR were retained as linear terms. Influential observations were assessed using Cook’s distance. No observation had a Cook’s distance greater than 1. A sensitivity analysis was performed by repeating the fully adjusted model after excluding the observation with the highest Cook’s distance to assess whether the findings were dependent on a single influential observation.

No missing data were observed for the variables included in the analyses, and all available data were included in the primary analyses.

No formal sample size calculation was performed due to the exploratory pilot nature of the study. Given the exploratory pilot design and limited sample size, all multivariable analyses were considered hypothesis-generating and should be interpreted cautiously. The study was not powered for definitive inference but to estimate effect sizes and assess feasibility for future larger studies. Accordingly, interpretation emphasized effect estimates and 95% confidence intervals rather than statistical significance alone.

All statistical tests were two-sided, and a p value < 0.05 was considered statistically significant. Exact p values are reported where appropriate. All statistical analyses were performed using Jamovi software (version 2.7; The Jamovi Project, Sydney, Australia), which is based on the R statistical environment.

## Results

### Participant characteristics

A total of 41 patients with type 2 diabetes mellitus (T2DM) were included, comprising 20 patients with diabetic peripheral neuropathy (DPN) and 21 patients without neuropathy (NoDPN). Clinical and demographic characteristics of both groups are summarized in Table 1. Although median eGFR was lower in the DPN group than in the NoDPN group, the between-group difference was not statistically significant (71.10 [IQR 49.65–84.15] vs 79.80 [IQR 63.00–90.00] mL/min/1.73 m^2^; p = 0.081).

**Table 1:** Clinical and demographic characteristics of patients with type 2 diabetes mellitus with and without diabetic peripheral neuropathy.

Characteristics	DPN (n = 20)	NoDPN (n = 21)	p-value
Plasma NfL (pg/mL)	12.96 (8.78–21.75)	8.29 (5.16–13.27)	**0.035**
Age (years)	70.00 (65.75–76.50)	68.00 (57.00–76.00)	0.441
Sex (male), n (%)	12 (60%)	11 (52%)	0.58
Duration T2DM (years)	17.00 (8.75–22.75)	5.00 (2.00–10.00)	**<0.001**
Body mass index (kg/m²)	30.30 (25.62–34.33)	31.22 (29.07–36.16)	0.315
HbA1c (%)	7.80 (7.19–9.78)	6.38 (6.21–6.70)	**<0.001**
eGFR (mL/min/1.73 m²)	71.10 (49.65–84.15)	79.80 (63.00–90.00)	0.081
Total cholesterol (mmol/L)	4.16 (3.70–4.78)	4.40 (3.64–4.68)	0.825
HDL cholesterol (mmol/L)	1.31 (1.15–1.56)	1.20 (1.04–1.62)	0.705
LDL cholesterol (mmol/L)	2.23 (2.00–2.82)	2.31 (1.97–2.95)	0.774
Triglycerides (mmol/L)	1.58 (1.01–2.58)	1.34 (1.06–1.89)	0.845
Metformin use, n (%)	16 (80%)	18 (85.7%)	0.697
Other oral antidiabetics, n (%)	13 (65%)	5 (23.8%)	**0.008**
Insulin therapy, n (%)	14 (70%)	1 (4.8%)	**<0.001**
Statins, n (%)	18 (90%)	13 (61.9%)	0.067

Data are presented as median (interquartile range, IQR) or n (%), as appropriate. Between-group comparisons were performed using the Mann–Whitney U test for continuous variables and the χ^2^ test or Fisher’s exact test for categorical variables, as appropriate.

DPN, diabetic peripheral neuropathy; NoDPN, type 2 diabetes mellitus without diabetic peripheral neuropathy; NfL, neurofilament light chain; BMI, body mass index; HbA1c, glycated hemoglobin; eGFR, estimated glomerular filtration rate; HDL, high-density lipoprotein; LDL, low-density lipoprotein.

### Differences in plasma NfL concentrations

**Fig 1:**
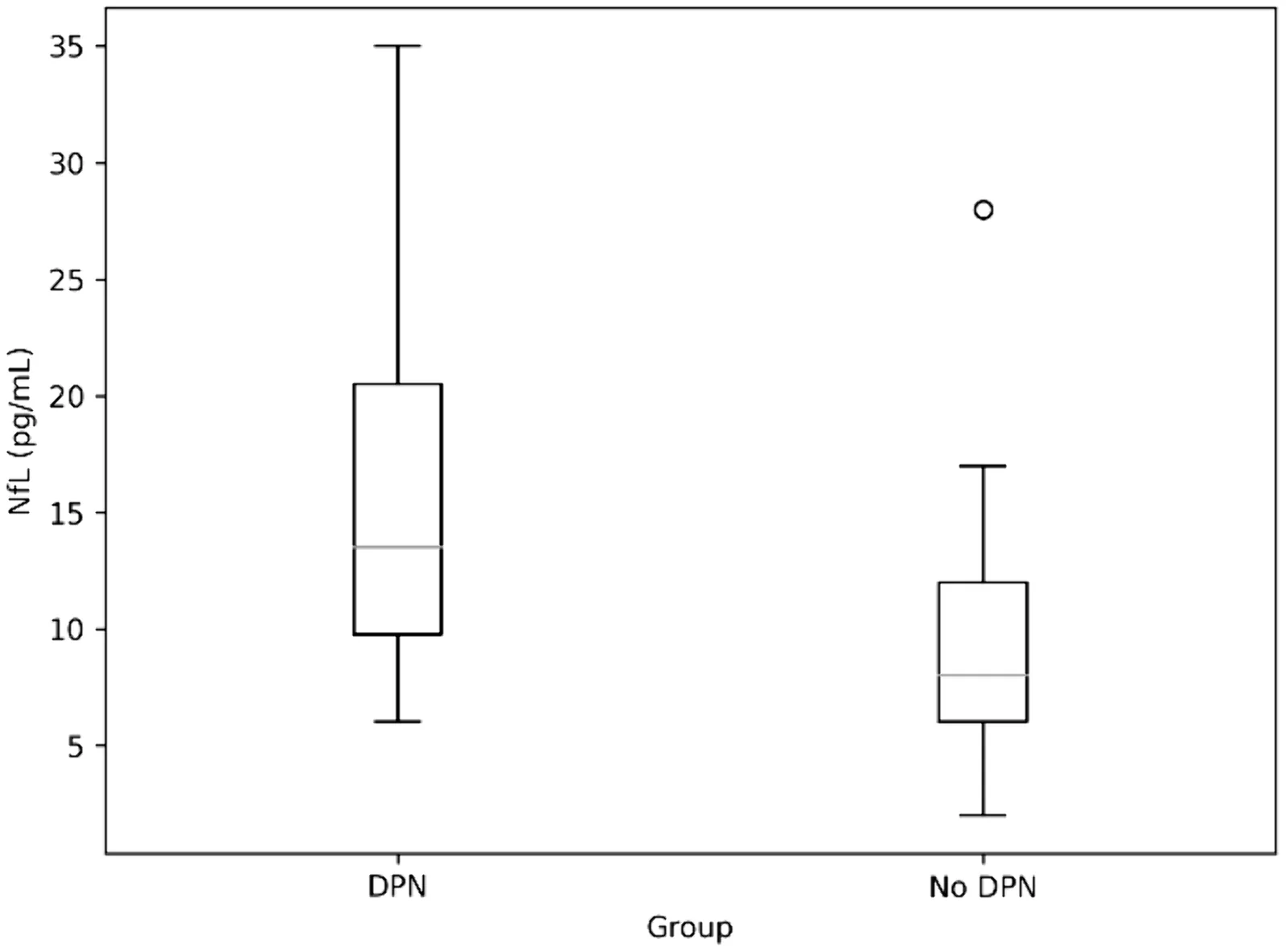
Plasma neurofilament light chain concentrations in patients with and without diabetic peripheral neuropathy. Boxplots showing plasma NfL concentrations in patients with diabetic peripheral neuropathy (DPN) and those without neuropathy (NoDPN). Boxes represent the interquartile range (IQR), with the median indicated by the central line. Whiskers indicate the range excluding outliers.

Plasma neurofilament light chain (pNfL) concentrations were significantly higher in patients with DPN compared with those without neuropathy (median 12.96 pg/mL [IQR 8.78–21.75] vs 8.29 pg/mL [IQR 5.16–13.27], p = 0.035) (Fig 1).

### Correlations between NfL and clinical variables

**Fig 2:**
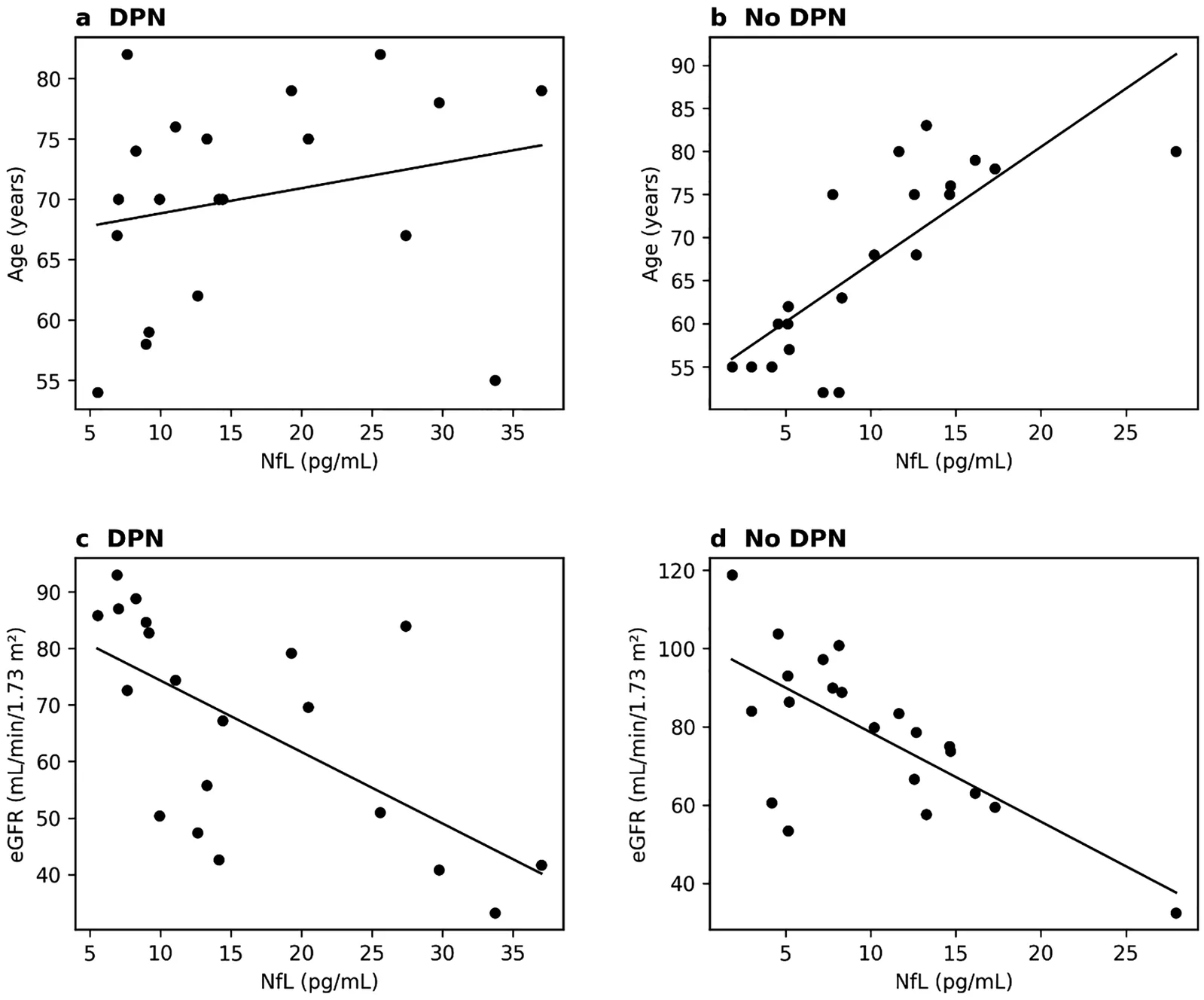
Associations of plasma neurofilament light chain concentrations with age and renal function. Scatter plots illustrating the associations of plasma NfL concentrations with age and estimated glomerular filtration rate (eGFR): (a) age in patients with diabetic peripheral neuropathy (DPN), (b) age in patients without neuropathy (NoDPN), (c) eGFR in patients with DPN, and (d) eGFR in patients without neuropathy (NoDPN).

In the exploratory group-stratified analyses, plasma NfL concentrations were inversely correlated with eGFR in both the DPN (ρ = −0.73, p < 0.001) and NoDPN groups (ρ = −0.60, p = 0.004) (Table 2 and Fig 2). NfL was positively correlated with age in the NoDPN group (ρ = 0.81, p < 0.001), whereas the corresponding correlation in the DPN group was weaker and not statistically significant (ρ = 0.32, p = 0.16). No formal statistical comparison of correlation coefficients between groups was performed. Additional group-stratified correlations between NfL and clinical variables are reported in Table 2.

**Table 2:** Spearman’s rank correlations between plasma neurofilament light chain and clinical variables in patients with and without diabetic peripheral neuropathy.

Characteristics	DPN (n = 20) Spearman’s ρ	p-value	NoDPN (n = 21) Spearman’s ρ	p-value
Age (years)	+0.324	0.163	+0.812	**<0.001**
Sex (male = 1 female = 0)	+0.241	0.320	+0.063	0.800
Duration T2DM (years)	+0.276	0.239	+0.311	0.169
Body mass index (kg/m²)	−0.088	0.712	−0.088	0.703
HbA1c (%)	−0.056	0.816	−0.384	0.086
eGFR (mL/min/1.73 m²)	−0.734	**<0.001**	−0.604	**0.004**
Total cholesterol (mmol/L)	−0.294	0.208	+0.170	0.459
HDL cholesterol (mmol/L)	+0.021	0.932	+0.770	**<0.001**
LDL cholesterol (mmol/L)	−0.471	**0.036**	−0.040	0.863
Triglycerides (mmol/L)	−0.060	0.801	−0.223	0.330

Spearman’s rank correlation coefficients (ρ) are presented. No correction for multiple comparisons was applied because of the exploratory nature of the study.

DPN, diabetic peripheral neuropathy; NoDPN, type 2 diabetes mellitus without diabetic peripheral neuropathy; T2DM, type 2 diabetes mellitus; NfL, neurofilament light chain; BMI, body mass index; HbA1c, glycated hemoglobin; eGFR, estimated glomerular filtration rate; HDL, high-density lipoprotein; LDL, low-density lipoprotein.

### Sequential linear regression analysis

Sequential linear regression models with ln(NfL) as the dependent variable were used to examine the association between DPN status and plasma NfL concentrations before and after adjustment for age and eGFR.

In Model 1, DPN status was associated with higher ln(NfL) concentrations (B = 0.499, 95% CI 0.108 to 0.890; p = 0.014). The model explained 14.6% of the variance in ln(NfL) (R^2^ = 0.146, adjusted R^2^ = 0.124; F(1, 39) = 6.65, p = 0.014). After adjustment for age in Model 2, the association between DPN status and ln(NfL) was attenuated but remained statistically significant (B = 0.383, 95% CI 0.061 to 0.704; p = 0.021). Age was positively associated with ln(NfL) (B = 0.038, 95% CI 0.021 to 0.055; p < 0.001). Model 2 explained 45.3% of the variance in ln(NfL) (R^2^ = 0.453, adjusted R^2^ = 0.424; F(2, 38) = 15.72, p < 0.001). In the fully adjusted Model 3, additional adjustment for eGFR further attenuated the association between DPN status and ln(NfL), which was no longer statistically significant (B = 0.241, 95% CI −0.040 to 0.522; p = 0.091). Age remained positively associated with ln(NfL) (B = 0.025, 95% CI 0.010 to 0.041; p = 0.002), whereas eGFR was inversely associated with ln(NfL) (B = −0.015, 95% CI −0.023 to −0.008; p < 0.001). The fully adjusted model explained 62.0% of the variance in ln(NfL) (R^2^ = 0.620, adjusted R^2^ = 0.589; F(3, 37) = 20.08, p < 0.001) (Table 3).

**Table 3:** Sequential linear regression models of factors associated with natural log-transformed plasma neurofilament light chain concentrations.

Model characteristic	Model 1, B(95% CI)	p-value	Model 2, B(95% CI)	p-value	Model 3, B(95% CI)	p-value
DPN status (ref.: NoDPN)	0.499(0.108 to 0.890)	**0.014**	0.383(0.061 to 0.704)	**0.021**	0.241(−0.040 to 0.522)	0.091
Age (years)	—	—	0.038(0.021 to 0.055)	**<0.001**	0.025(0.010 to 0.041)	**0.002**
eGFR (mL/min/1.73 m²)	—	—	—	—	−0.015 (−0.023 to −0.008)	**<0.001**
R²	0.146	—	0.453	—	0.620	—
Adjusted R²	0.124	—	0.424	—	0.589	—
Overall F-test	F(1, 39) = 6.65	**0.014**	F(2, 38) = 15.72	**<0.001**	F(3, 37) = 20.08	**<0.001**
N	41	—	41	—	41	—

The dependent variable was natural log-transformed plasma neurofilament light chain concentration [ln(NfL)]. Model 1 included DPN status. Model 2 included DPN status and age. Model 3 included DPN status, age, and eGFR. NoDPN was the reference category for DPN status. B represents the unstandardized regression coefficient; 95% CI represents the 95% confidence interval.

DPN, diabetic peripheral neuropathy; NoDPN, type 2 diabetes mellitus without diabetic peripheral neuropathy; NfL, neurofilament light chain; eGFR, estimated glomerular filtration rate.

Additional analyses provided no evidence of substantial non-linearity in the associations of age or eGFR with ln(NfL). The centered quadratic terms for age and eGFR were not statistically significant (p = 0.914 and p = 0.810, respectively), and their joint inclusion did not improve model fit (partial F-test: F(2, 35) = 0.044, p = 0.957).

Model diagnostics showed no evidence of substantial multicollinearity (all VIFs ≤ 1.32). Residuals of the fully adjusted model were compatible with normality (Shapiro–Wilk W = 0.981, p = 0.732), and inspection of the residual-versus-fitted and Q–Q plots did not indicate major violations of model assumptions. The highest Cook’s distance was 0.447. In a sensitivity analysis excluding the observation with the highest Cook’s distance, the direction and statistical significance pattern of the associations remained unchanged. DPN status remained non-significant (B = 0.211, 95% CI −0.058 to 0.479; p = 0.120), whereas age (B = 0.033, 95% CI 0.017 to 0.049; p < 0.001) and eGFR (B = −0.011, 95% CI −0.019 to −0.003; p = 0.007) remained significantly associated with ln(NfL) (Table 4).

**Table 4:** Sensitivity analysis of the fully adjusted linear regression model after exclusion of the observation with the highest Cook’s distance.

Model characteristic	Primary fully adjusted modelB (95% CI)	p-value	Sensitivity analysisB (95% CI)	p-value
DPN status (ref.: NoDPN)	0.241 (−0.040 to 0.522)	0.091	0.211 (−0.058 to 0.479)	0.120
Age, years	0.025 (0.010 to 0.041)	**0.002**	0.033 (0.017 to 0.049)	**<0.001**
eGFR, mL/min/1.73 m²	−0.015 (−0.023 to −0.008)	**<0.001**	−0.011 (−0.019 to −0.003)	**0.007**
R²	0.620	—	0.639	—
Adjusted R²	0.589	—	0.608	—
N	41	—	40	—

The dependent variable was natural log-transformed plasma neurofilament light chain concentration [ln(NfL)]. Both models included DPN status, age, and eGFR. NoDPN was the reference category for DPN status. The sensitivity analysis excluded the observation with the highest Cook’s distance in the primary fully adjusted model (Cook’s D = 0.447). B represents the unstandardized regression coefficient; 95% CI represents the 95% confidence interval.

DPN, diabetic peripheral neuropathy; NoDPN, type 2 diabetes mellitus without diabetic peripheral neuropathy; NfL, neurofilament light chain; eGFR, estimated glomerular filtration rate.

## Discussion

In this pilot cross-sectional study, we evaluated circulating NfL in individuals with type 2 diabetes mellitus (T2DM) with and without diabetic peripheral neuropathy (DPN). Patients with DPN had higher plasma NfL concentrations than patients without neuropathy. In sequential regression analyses, DPN status was associated with higher ln(NfL) concentrations in the unadjusted model and remained associated after adjustment for age. After additional adjustment for eGFR, the estimated association was further attenuated and no longer statistically significant, whereas age and eGFR remained associated with ln(NfL). These findings indicate that the association between DPN status and circulating NfL is sensitive to adjustment for renal function and should be interpreted cautiously given the exploratory design and limited sample size.

In unadjusted between-group comparisons, we observed statistically significant differences between patients with DPN and diabetic controls without neuropathy (NoDPN), not only in plasma NfL concentrations but also in HbA1c levels and diabetes duration. Patients with DPN had significantly higher NfL concentrations, poorer long-term glycemic control, and longer T2DM duration compared with participants without neuropathy. Although these findings are compatible with the established contribution of chronic metabolic disturbances to DPN pathophysiology [24–28], HbA1c and diabetes duration were not included in the sequential regression models because the number of explanatory variables was deliberately restricted given the small sample size. Their potential contribution to circulating NfL concentrations therefore cannot be determined from the present multivariable analyses.

The sequential regression models provide a more nuanced interpretation of the between-group difference in circulating NfL. Adjustment for age attenuated the regression coefficient for DPN status, although the association remained statistically significant. After additional adjustment for eGFR, the estimated association was further attenuated and its 95% CI included zero. Thus, the fully adjusted model should not be interpreted as evidence that circulating NfL is associated with renal function rather than DPN or as evidence of the absence of an association between DPN and NfL. Instead, the results indicate that the estimated association between DPN status and circulating NfL is reduced when renal function is taken into account. Given the limited sample size and the resulting uncertainty of the effect estimates, these findings should be considered hypothesis-generating. The sensitivity analysis yielded the same qualitative pattern of associations after exclusion of the observation with the highest Cook’s distance, suggesting that the findings of the fully adjusted model were not dependent on this single observation.

An important finding of this study was the strong inverse association between NfL concentrations and renal function. This observation is in line with previous reports indicating that impaired renal function is associated with higher circulating NfL levels [17–19,28]. Although the precise mechanisms underlying this relationship remain incompletely understood, several plausible explanations have been proposed. One hypothesis suggests that NfL is at least partially cleared from the circulation via renal pathways [18]. Alternatively, reduced renal function may indirectly influence neuroaxonal integrity through alterations in erythropoietin and vitamin D metabolism, both of which have been implicated in neuroprotection [18,29].

Consistent with our regression findings, eGFR remained inversely associated with ln(NfL) after adjustment for DPN status and age. Importantly, aging and renal function are biologically interrelated, as increasing age is generally associated with a decline in glomerular filtration rate. Consequently, age-related differences in circulating NfL concentrations may partly reflect age-associated reductions in renal function and the potential influence of impaired renal clearance on circulating NfL levels. Previous reference-cohort studies have identified age as a major determinant of circulating NfL concentrations and have also demonstrated contributions from BMI and renal function, with the influence of impaired kidney function becoming particularly relevant at lower eGFR levels [17]. In the present sequential regression analyses, age remained positively associated with ln(NfL) after adjustment for eGFR, whereas eGFR remained inversely associated with ln(NfL) after adjustment for age and DPN status, suggesting that both factors contributed to NfL variability in this cohort.

The role of renal function should nevertheless be interpreted cautiously. The present cross-sectional data cannot determine whether eGFR acts as a conventional confounder, whether reduced renal clearance directly contributes to higher circulating NfL concentrations, or whether renal dysfunction and neuroaxonal injury partly reflect shared diabetes-related systemic processes. Accordingly, our findings demonstrate an association between renal function and circulating NfL but do not establish the causal pathway underlying this relationship.

Age represents another key determinant of NfL variability. In our study, a positive association between NfL and age was observed in participants without neuropathy, whereas this relationship did not reach statistical significance in the DPN group. In larger cohorts, age-dependent increases in NfL have been consistently demonstrated [13–15,27,30–32], likely reflecting cumulative neuroaxonal damage over time. However, the difference in statistical significance between the two group-stratified correlations does not establish that the underlying associations differ between groups, and no formal comparison of correlation coefficients was performed. The sequential regression analyses further showed a positive association between age and ln(NfL) after adjustment for DPN status and after additional adjustment for eGFR.

In addition to renal function and age, we observed associations between NfL and lipid parameters, including LDL and HDL cholesterol. Because multiple correlations were examined without correction for multiple testing and the subgroup sample sizes were small, these findings should be considered exploratory and hypothesis-generating rather than evidence of group-specific biological relationships. Nevertheless, previous studies support a potential contribution of broader cardiometabolic factors to neuroaxonal injury in T2DM [33,34], and these associations warrant further investigation in larger cohorts.

Several limitations should be acknowledged. First, the relatively small sample size limits statistical power, particularly for detecting small effects. The small sample size also limited the precision of regression estimates and restricted the number of explanatory variables that could reasonably be included in the models. Second, DPN was not assessed using standardized scoring systems, which may have introduced heterogeneity. In addition, the requirement for electrophysiological abnormalities restricts the generalizability of the findings to patients meeting this diagnostic definition and does not capture the full spectrum of diabetic neuropathy, including small-fiber neuropathy. Because the neurological and electrophysiological assessments were performed as part of routine clinical care before study enrollment, detailed information on the individual nerves examined, specific sensory testing modalities, nerve-specific electrophysiological values, and procedural aspects of NCS, including stimulation and recording distances and limb temperature monitoring, was unavailable for retrospective analysis. This limited methodological reproducibility, precluded a more detailed characterization of neuropathy severity, and prevented assessment of the potential influence of specific electrophysiological abnormalities on circulating NfL concentrations. Third, the cross-sectional design precludes causal inference. Fourth, the study did not include a healthy control group or age-adjusted NfL z-scores derived from an external reference population; therefore, it was not possible to determine whether individual NfL concentrations were elevated relative to age-specific reference values. Fifth, residual confounding by factors not included in the parsimonious regression models, including BMI, glycemic control, diabetes duration, other diabetes-related complications, and unrecognized neurological comorbidities, cannot be excluded. Finally, although model diagnostics and the sensitivity analysis supported the qualitative stability of the fully adjusted model, these analyses do not overcome the limited statistical precision associated with the small sample size.

## Conclusion

In this pilot cross-sectional study, DPN status was associated with higher plasma NfL concentrations in unadjusted and age-adjusted analyses, whereas the estimated association was attenuated and no longer statistically significant after additional adjustment for eGFR. Age and eGFR remained associated with ln(NfL) in the fully adjusted model. These exploratory findings highlight the importance of considering age and renal function when interpreting circulating NfL concentrations in T2DM and require validation in larger prospective studies.

## Abbreviations

BMI: body mass index
CI: confidence interval
DPN: diabetic peripheral neuropathy
eGFR: estimated glomerular filtration rate
HbA1c: glycated hemoglobin
HDL: high-density lipoprotein
LDL: low-density lipoprotein
NCS: nerve conduction studies
NfL: neurofilament light chain
NoDPN: type 2 diabetes mellitus without diabetic peripheral neuropathy
T2DM: type 2 diabetes mellitus
